# Enhancement of carrier transport characteristic in the Sb_2_Se_2_Te topological insulators by N_2_ adsorption

**DOI:** 10.1038/s41598-017-05369-y

**Published:** 2017-07-11

**Authors:** Shiu-Ming Huang, Shih-Jhe Huang, Ching Hsu, Paritosh V. Wadekar, You-Jhih Yan, Shih-Hsun Yu, Mitch Chou

**Affiliations:** 1 Department of Physics, National Sun Yat-Sen University, Kaohsiung, 80424 Taiwan; 2 Department of Materials and Optoelectronic Science, National Sun Yat-Sen University, Kaohsiung, 80424 Taiwan; 3 Taiwan Consortium of Emergent Crystalline Materials, TCECM, Taipei, Taiwan

## Abstract

The carrier transport characteristics of Sb_2_Se_2_Te topological insulators were investigated, after exposure to different levels of nitrogen gas. The magnetoresistance (MR) slope for the Sb_2_Se_2_Te crystal increased by approximately 100% at 10 K after 2-days of exposure. The Shubnikov-de Haas (SdH) oscillation amplitude increased by 30% while oscillation frequencies remained the same. MR slopes and the mobilities had the same dependency on temperature over a wide temperature range. All measured data conformed to a linear correlation between MR slope and mobility, supporting our hypothesis that the MR increase and the SdH oscillation enhancement might be caused by mobility enhancement induced by adsorbed N_2_ molecular.

## Introduction

Topological insulators (TIs) are characterized by their distinctive surface states with linear dispersion, and it exhibits remarkable helical spin texture^1–7^. The spin-momentum locking effect and various transport and optic characteristics of the surface states have been extensively investigated^8–23^. To optimize the transport and optic characteristics of the surface state, the Fermi level tuning through back-gate voltage^24, 25^, material component adjustment^25–30^ are widely used. Various studies support that these fantastic characteristics are strongly related to the carrier mobility. Thus, as well as these complicating artificial techniques, an appropriate treatments to enhance the carrier mobility might be efficient way to optimize these carrier transport characteristics.

It is reported that the adsorbed molecules and/or extrinsic impurities and defects on TI surfaces might bend the band structure. The band bending near the surface of topological insulators may lead to a highly mobile two-dimensional electron gas (2DEG)^31^. This induced 2DEG will increase effective carrier mobility. This effect is experimentally observed in the Bi_2_Se_3_ topological insulator^31^. On the other hand, it was reported that nitrogen-doping enhanced the SdH oscillation amplitude in graphene and the amplitude enhancement increased when the nitrogen-doping rate was increased^32^. Theoretical studies have proposed that surface state carriers are robust against structural defects and extrinsic impurities because of their strong spin-orbit interaction^1–7^. Thus, it might be an appropriate way to enhance the carrier mobility through physical molecular adsorption on the surface of topological insulators. Many reports have focused on the influence of the surface chemical/physical interaction on TI in various kinds of gas environments through ARPES^33^ and XPS^33–35^. However, the similar effect on these transport characteristics of TI are still rare^17, 36^.

In this work, we experimentally perform the electrical transport characteristics in the Sb_2_Se_2_Te with different levels of N_2_ exposure. The results reveal that the non-saturating magnetoresistance (MR) ratio increased by approximately 100% at 10 K and the quantum Shubnikov-de Haas (SdH) oscillation amplitude increased by 30% after 2-days of exposure. The carrier scattering time and mobility increased by 30%. This results suggested that nitrogen-adsorbed on the surface may have optimized the carrier transport in the Sb_2_Se_2_Te topological insulators.

## Experimental Methods

Single crystals of Sb_2_Se_2_Te were grown using a homemade resistance-heated floating zone furnace (RHFZ). The raw materials used to make the Sb_2_Se_2_Te crystals were mixed according to the stoichiometric ratio. At first, the stoichiometric mixtures of high purity elements Sb (99.995%), Se (99.995%) and Te (99.995%) was melted at 700~800 °C for 20 h and slowly cooled to room temperature in an evacuated quartz tube. The resultant material was then used as a feeding rod for the RHFZ experiment. Our previous work demonstrated that TI with extremely high uniformity can be obtained using the RHFZ method. After growth, the crystals were furnace cooled to room temperature. The as-grown crystals were cleaved along the basal plane, using a silvery reflective surface, and then prepared for the further experiments. Energy-dispersive X-ray spectroscopy (EDS) confirmed that the crystals contained Sb:Se:Te = 2:2:1, and the XRD spectrum of the crystal was consistent with the Sb_2_Se_2_Te database.

The cleaved Sb_2_Se_2_Te single crystals were obtained using the Scotch-tape method. The thickness of the crystals was approximately 100 *μ*m and their surface was approximately 6-mm × 5-mm. Gold wires were electrically attached to the cleaved crystal surface using a silver paste. Magnetotransport measurements were performed using the standard six-probe technique in a commercial apparatus (Quantum Design PPMS) with a magnetic field of up to 9 T. The magnetic field was applied perpendicular to the large cleaved surface.

## Results and Discussion

**Figure 1:**
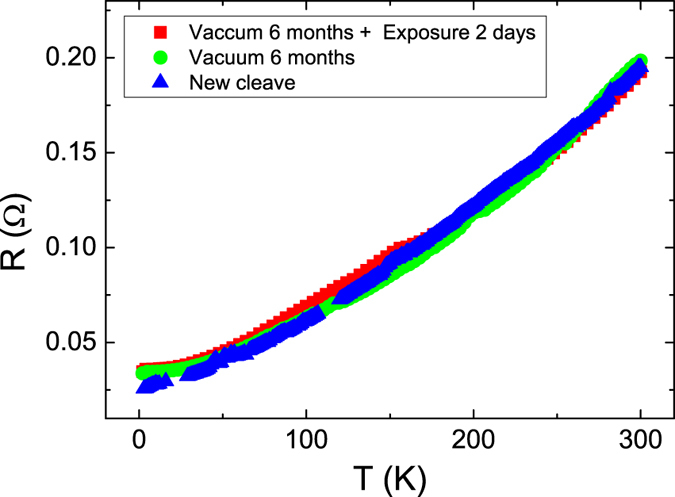
The temperature dependent resistance of newly cleaved, 6-months in vacuum, 2-days N_2_ atmosphere exposed crystal. Metallic behavior was observed, and the residual resistance ratio was 5. Because resistances have the same temperature dependence. N_2_ exposure time does not obviously influence the overall characteristics of the TI.

To determine the influence of the N_2_ exposure on the intrinsic transport characteristics, experiments were performed on the same sample under different levels of N_2_ exposure. Figure 1 illustrates the temperature dependence of the TI’s resistance for the newly cleaved crystal, the crystal after 6-months in a vacuum (N_2_ at 0.1 Torr), and the crystal subsequently exposed to N_2_ for 2-days at 1 atm. The measured temperature dependent resistances are similar for all three exposure levels with the only slight change at low temperatures. That implies that no chemical reaction but only physical molecular adsorption during the N_2_ exposure. Resistance decreased as temperature decreased, which is metallic behavior. The residual resistance ratio was approximately 5, which indicated that the sample was of superior quality and the N_2_ exposure did not substantially damage the quality of the sample. Residual resistance at low temperature is strongly related to sample defects and impurities. Figure 1 illustrates that larger residual resistance was detected in crystals exposed to N_2_ for longer duration. Thus, more N_2_ molecules were adsorbed on the Sb_2_Se_2_Te surface after extended N_2_ exposure.

**Figure 2:**
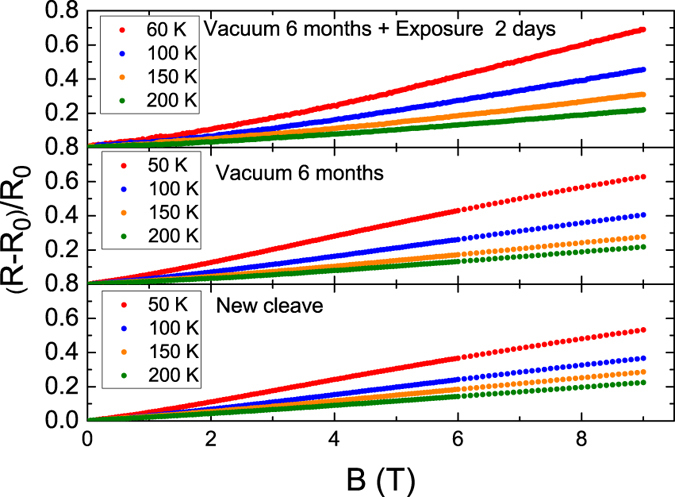
Temperature dependence of MR slope for newly cleaved, 6-months in vacuum, and 2-days N_2_ exposed samples. The MR slope increases as temperature decreases. N_2_ exposure does not completely smear out the non-saturated magnetoresistance. Smaller MR slopes are seen at longer exposure times.

Figure 2 illustrates the MR ratio (*R*(*B*) − *R*(*B* = 0))/*R*(*B* = 0), of the newly cleaved, 6-months in vacuum, and 2-days N_2_ exposure sample. Non-saturating MR was observed at all levels of N_2_ exposure, and no obvious saturation tendency was observed for magnetic fields of up to 9 T over a wide temperature ranges. Higher MR ratios were discovered at lower temperatures and longer N_2_ exposure times. The MR ratio increased from 0.55 for the newly cleaved sample to 0.7 for the sample exposed to N_2_ for 2 days at approximately 50 K and 9 T. Thus, N_2_ exposure enhanced the MR ratio.

Parish and Littlewood developed a classical random-resistor model (PL model) to explain the behavior of the linear MR^37^. It proposed that the linear MR originates from the mobility fluctuation in highly disordered or weakly disordered high-mobility materials. The local current density acquires spatial fluctuations in both magnitude and direction in an inhomogeneous carrier and mobility distribution. Therefore, it causes fluctuation in the Hall field that contributes to the linear MR, and this MR is determined by the mobility fluctuations rather than the mobility in weakly disordered conditions. Based on this PL model, the slope of the linear MR is proportional to the mobility in the weak mobility fluctuation region, Δ*μ*/〈*μ*〉 < 1, where 〈*μ*〉 is the average mobility and Δ*μ* is the mobility fluctuation level. The carrier mobility, *μ*, could be determined through the relation $\mu =\frac{1}{neR}$, where *n* and *e* are the carrier concentration and carrier charge, respectively. The carrier concentration is determined from the Hall resistance.

**Figure 3:**
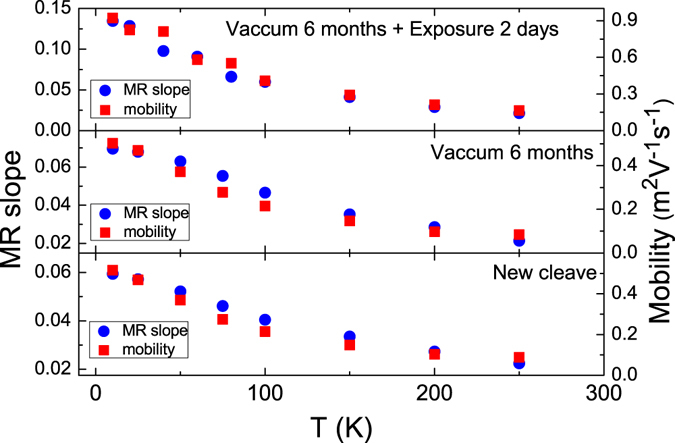
The MR slope and mobility, with the same temperature dependence, for different N_2_ exposure times. The MR slope and mobility had the same temperature dependence over a wide temperature range for different levels of N_2_ exposure. The MR slope and mobility increased approximately 100% after 2-days N_2_ exposure.

Herein, we define the MR slope as the slope of linear MR extracted from fitting the linear MR at high magnetic fields. Figure 3 shows that the MR slope and *μ* had the same temperature dependence over a wide temperature range for different levels of N_2_ exposure. This shows excellent consistency with the prediction of the PL model in the weak mobility fluctuation region. The MR slope (mobility) increased from 0.06 (0.45 *m* ^2^/*V* · *s*) for the newly cleaved sample to 0.15 (0.9 *m* ^2^/*V* · *s*) for the 2-days N_2_ exposed sample at 10 K. Both the MR slope and mobility roughly doubled at 10 K after 2-days N_2_ exposure, which is consistent with the theoretical prediction that the MR is proportional to the mobility. Thus, all the observed MR slope changes may have been directly related to the mobility changes under the same condition in the weak mobility fluctuation region.

**Figure 4:**
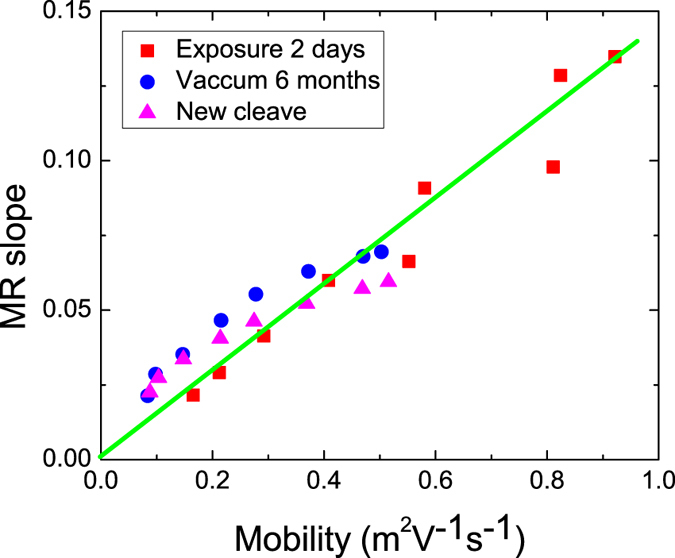
The MR slope as a function of the mobility. The measured data collapses onto one line, demonstrating that the MR slope is proportional to the mobility.

Figure 4 plots the MR slope of the measured data as a function of the mobilities for the same sample after different levels of N_2_ exposure at different temperatures. Linear correlation between MR slope and mobility was demonstrated over a wide mobility range (*μ* = 0.1–1.0 m^2^/*V* · *s*). N_2_ exposure induced spatial mobility disorder, led to carrier domination, and was a possible source of the decreased mobility observed. As illustrated in Fig. 4, the MR slope was proportional to the mobility, which supports the hypothesis that the enhanced linear MR ratio was caused by mobility enhancement.

Theoretical calculations proposed that extrinsic defects and impurities on the crystal surface might lead to electronic band bending, and downward band bending at the surface would induce a 2DEG. This effect was experimentally observed using ARPES in the Bi_2_Se_3_ TI with adsorbed N_2_ molecules on the surface. The induced 2DEG was formed at the surface and coexisted with the TI surface state shortly after the Bi_2_Se_3_ was exposed to N_2_ gas^31^. A 2DEG induced by extrinsic impurities contributed high-mobility carriers to the system and thus increased the effective mobility of the total carrier transport. Furthermore, following the PL model, the non-saturating linear MR originates from mobility fluctuations induced by the structural defects. This is consistent with the mobility-enhancement mechanism. Thus, the observed enhancement of the linear MR may have been caused by the physical molecular adsorption of N_2_ on the surface. The experimental report shows that the large non-saturating MR that is observed in the Sb_2_Se_3_ with extremely high carrier mobility that is induced by the molecular adsorption on the surface^38^.

**Figure 5:**
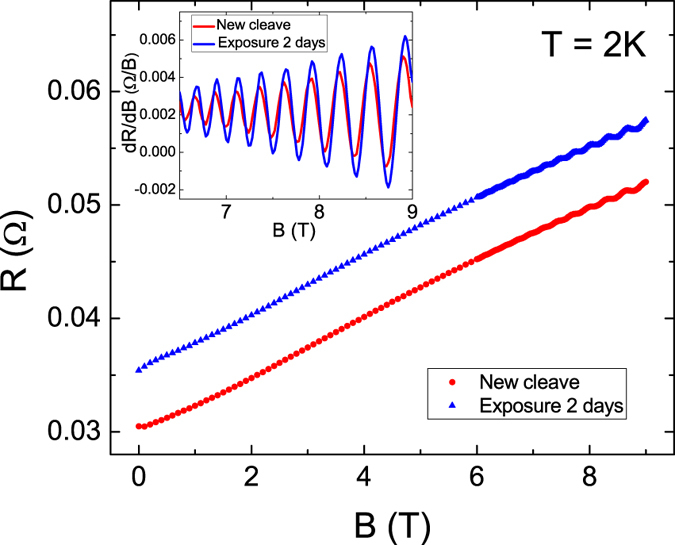
Magnetoresistances of newly cleaved and air exposed Sb_2_Se_2_Te crystal. Inset differential of the magnetoresistance at high magnetic fields. Larger oscillation amplitude was observed after N_2_ exposure.

To further confirm the observed enhanced linear MR ratio originates from the enhance carrier mobility that is induced by 2DEG. The SdH oscillation, widely known to be dominated by 2D carriers, is studied. Figure 5 illustrates the magnetoresistances of Sb_2_Se_2_Te crystals newly cleaved and after two days of exposure to atmosphere. The inset displays the differential of the magnetoresistance, and clear SdH oscillations are revealed. Previous experimental studies have reported that SdH oscillations disappear after TIs are exposed to the atmosphere for several hours, but we discovered that the oscillation amplitude increased by 30% after two days of N_2_ exposure.

**Figure 6:**
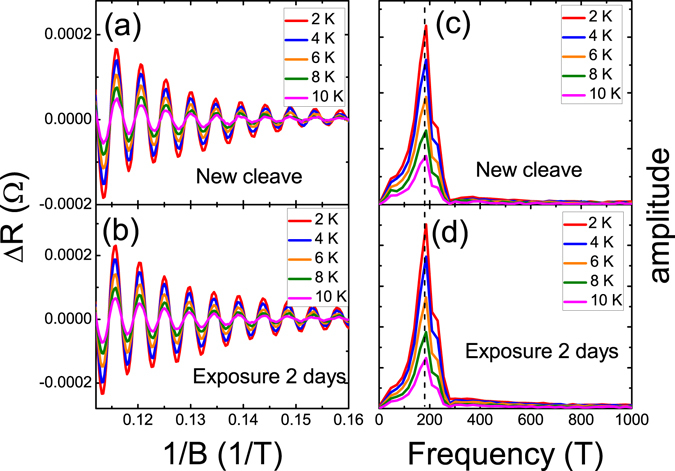
SdH oscillation as a function of inverse magnetic field and the related oscillation frequency. The oscillation amplitude increased by 30% after N_2_ exposure, whereas the oscillation frequencies remained the same.

Figure 6(a,b) show Δ*R*(*B*) as a function of 1/*B* for the newly cleaved crystal and the crystal after two days of N_2_ exposure, respectively. The Δ*R*(*B*) was the measured *R* after subtracting the smooth polynomial background function. The oscillation amplitudes increased after N_2_ exposure and the oscillation amplitudes decreased as the temperature increased. The oscillation periods were equal for all measured temperatures. The oscillation frequencies were extracted from the Fourier transform, and a major peak at frequency of *F* = 186 T was observed for all measured temperatures [Fig. 6(c,d)]. The SdH oscillation frequency, *F*, is directly related to the Fermi-level cross section through the Onsager relation $F=(\frac{\hslash c}{2e}){k}_{F}^{2}$, where *k*_*F*_ is the Fermi vector, *e* is the electron charge and *ħ* is the Planck constant. From the observed SdH oscillation frequency, *F* = 187 T, *k*_*F*_ = 7.5 × 10^6^ cm^−1^ was calculated, which is consistent with the value (*k*_*F*_ = 7.8 × 10^6^ cm^−1^) obtained when ARPES was used on a different batches of the same Sb_2_Se_2_Te crystal.

The enhanced SdH oscillation amplitudes were reported in GaN/AlGaN 2DEG exposed to UV illumination. The enhanced oscillation amplitudes come from the increasing carrier due to the UV illumination^39^. The extracted carrier concentration increased by approximately 6% at 10 K after 2-days N_2_ exposure, which is an order of magnitude smaller than the total mobility change, implying that the carrier domination was not the predominant cause of the increased mobility observed in our work. On the other hand, the enhanced SdH oscillation amplitudes are also observed in a N-doped graphene, and larger oscillation amplitude was observed in a higher N-doped graphene. The oscillation frequency changes as the N-doped rate, that might come from the carrier doping that leads to the shift of the Fermi level. That is different from our observation that the oscillation frequencies are the same.

It is worthy to pay attention on that the SdH oscillation peaks slightly shift to higher magnetic fields. This indicates the shift of the Berry phase and the extracted Berry phase was 0.5 and 0.48, respectively. This effect is reported in the thin Bi nanoribbons with different levels of air exposure^40^. It is known that the Berry phase is 0.5 and 0 in systems with linear and parabolic E-K dispersions, respectively. This Berry phase shift might come from the combination of the TI surface state with linear E-K dispersion and the 2DEG with parabolic E-K dispersion. The coexistence of the topological state and a 2DEG on the surface was observed in Bi_2_Se_3_^31^. These results all support the conclusion that the observed SdH oscillation originated from the surface states of the Sb_2_Se_2_Te.

**Figure 7:**
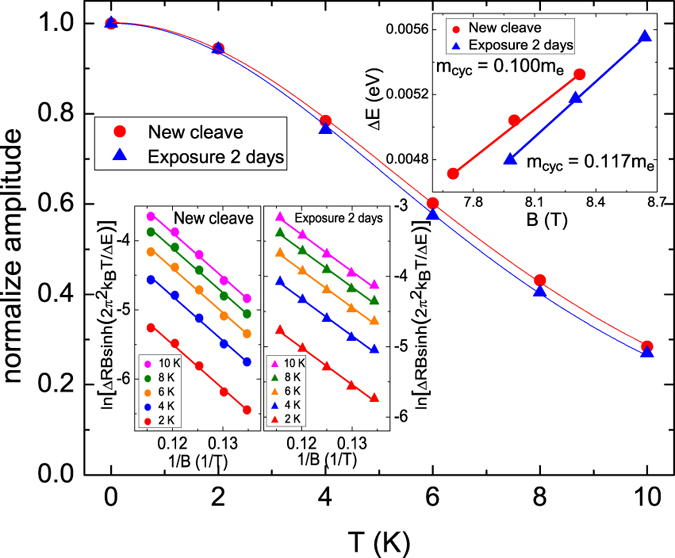
Normalize amplitudes as a function of temperature. The effective mass increased after N_2_ exposure.

The temperature dependence of SdH oscillation amplitudes is well described by Lifshitz-Kosevich theory, and can be expressed as

$${\rm{\Delta }}R(T,B)\propto \frac{\lambda (T/B)}{sinh(\lambda (T/B))}{e}^{-\lambda ({T}_{D})},$$

where $\lambda (T/B)=\mathrm{(2}{\pi }^{2}{k}_{B}T{m}_{cyc})/(\hslash eB)$, and the Dingle temperature ${T}_{D}=\hslash \mathrm{/(2}\pi {k}_{B}\tau )$. Using Lifshitz-Kosevich theory, the effective characteristic parameters of the transport carriers in the surface states of the TI can be extracted. Figure 7 illustrates the normalized oscillation amplitude at *B* = 8.7 T as a function of temperature after the two exposure times, and the results fit the Lifshitz-Kosevich prediction. Fitting determined that ${m}_{cyc}=0.100\,{m}_{0}$ and 0.117 *m*_0_ for the newly cleaved and two days exposed crystals, respectively, where *m*_0_ is the free electron mass. using the linear dispersion relation, ${v}_{F}=\hslash {k}_{F}/{m}_{cyc}$, the Fermi velocity of the carriers on the surface states was determined as *v*_*F*_ = 8.64 × 10^7^ cm/s and *v*_*F*_ = 7.40 × 10^7^ cm/s for the newly cleaved and two days exposed crystal, respectively.

The Dingle factor, ${e}^{-\lambda ({T}_{D})}$, is canceled out in a fitting using the normalized amplitude. Therefore, the Dingle temperature was extracted from a plot of oscillation amplitude as a function of inverse magnetic field. The Dingle temperature is related to the surface carrier lifetime through the relation ${T}_{D}=\hslash /(2\pi {k}_{B}\tau )$. The carrier transport characteristics of the surface states, such as the mean free path, ${l}_{2D}={v}_{f}\tau$, and carrier mobility, ${\mu }_{2D}=(e{l}_{2D})/(\hslash {k}_{F})$, were systematically determined and are listed in Table 1. The carrier life time, the carrier mobility and the carrier mass increase after 2-days N_2_ exposure.

**Table 1:** The extracted carrier characteristics of surface state in the same sample with different level of N_2_ exposure.

Characteristics	New cleave	N_2_ exposure
*τ* (s)	2.8 × 10^−14^	4 × 10^−14^
*ν*_*F*_ (*cm*/*s*)	8.6 × 10^7^	7.4 × 10^7^
*l*_2*D*_ (*cm*)	24 × 10^−7^	30 × 10^−7^
*μ*_2*D*_ (*m* ^2^/*Vs*)	0.0599	0.0766
*m*_*cyc*_	0.1 *m*_0_	0.117 *m*_0_

It comes to our attention that the SdH oscillation amplitude for the bismuth single crystals doped with a small amount of antimony is markedly enhanced with decreasing temperature^41^. It is proposed that the increasing electronic scattering time leads to the enhanced oscillation amplitudes. Our experimental results shows that the carrier scattering times increases after N_2_ exposure. That might be the intrinsic mechanism that leads to the enhanced mobility.

**Figure 8:**
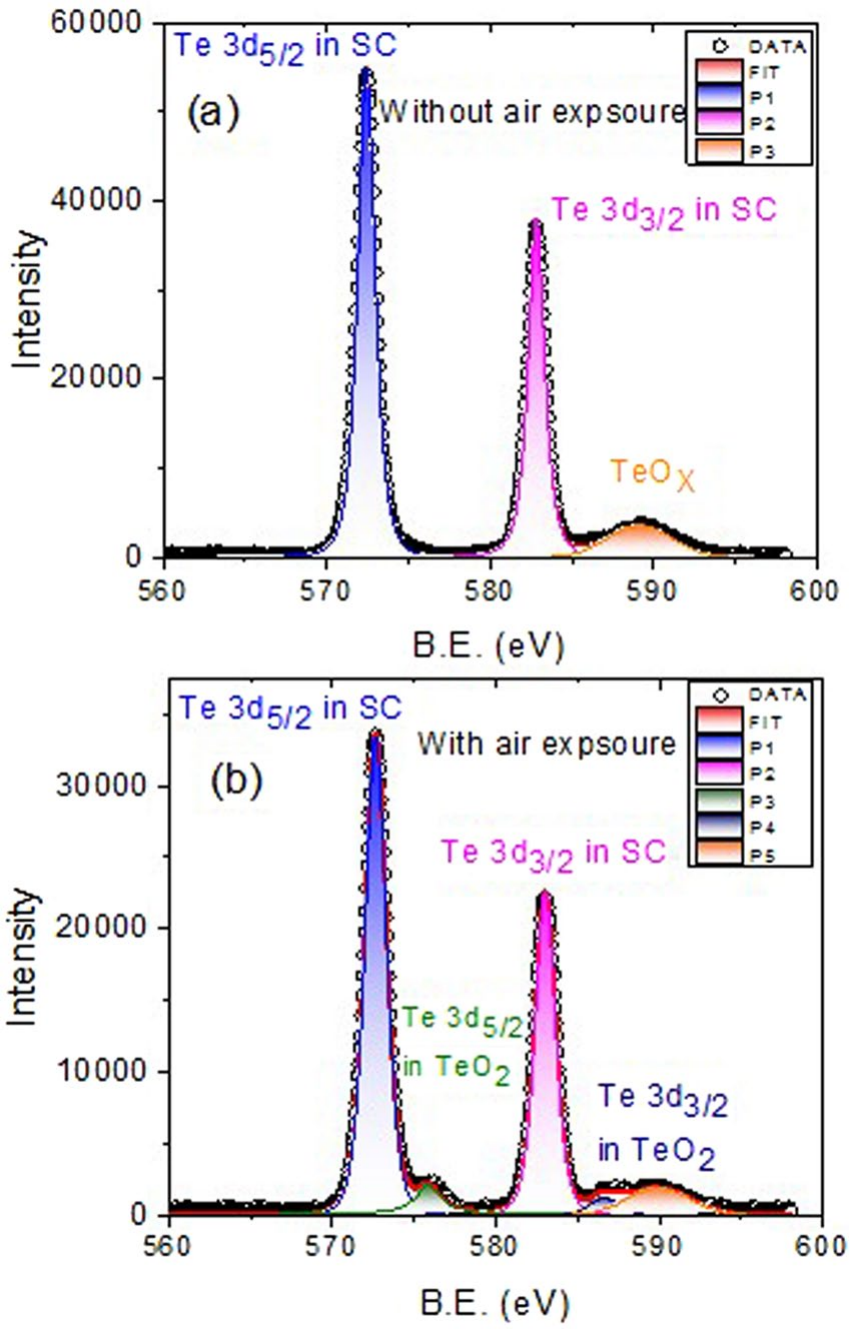
XPS results of the Te 3d scan, showing a small oxidation peak when the crystal was exposed to N_2_. The estimated oxidation layer thickness is 2.8 nm.

The surface states of TIs are theoretically tolerant to surface oxidation and scattering from structure defects and impurities due to spin-orbit interactions. However, all of previous experimental studies reported that surface oxidation suppressed carrier transport in the surface states. Surface oxidation was previously observed to smear out SdH oscillations after material was exposed to the atmosphere for several hours^35, 42^. Sb, Se, and Te are known to be oxidation activated, and to identify whether the oxidation and/or molecular adsorption occurred, X-ray photoelectron spectroscopy (XPS) were performed. The XPS results in Fig. 8(a,b) clearly reveal slight oxidation on the surface, and that the oxidation peaks appeared after N_2_ exposure. The thickness of the oxidation layer, *d*, was estimated from *I*_*o*_/*I*_*m*_, where *I*_*o*_ and *I*_*m*_ are the intensity of the sample with and without surface oxidation, respectively.

1 $$d=\lambda \,\cos (\theta )\mathrm{ln}[(1+\frac{{\rho }_{m}}{{\rho }_{o}})(\frac{{I}_{o}}{{I}_{m}})].$$

*λ*, *θ*, *ρ*_*m*_, and *ρ*_*o*_ are the mean electron escape depth, mean electron take off angle, atomic density of Te, and atomic density of TeO_2_, respectively^34, 43^. The estimated oxidation layer thickness was approximately 2.8 nm, consistent with its previously reported value^34^.

ARPES results have revealed that chemical oxidation and/or physical adsorption on the surface of TIs contributes to carrier doping and strongly shifts the Fermi level^33, 40^. As well as smearing out surface state transport behaviors, carrier doping limits the application potential of TIs. These are on-going problems and need to be solved before further application of these materials^36, 44^. Thus, the search for TI materials with surface states tolerant to oxidation, or techniques to prevent surface oxidation, is crucial. Our experimental result shows that the SdH oscillation frequencies of the Sb_2_Se_2_Te crystal were the same regardless of N_2_ exposure, which is consistent with theoretical predictions, and this resistance to surface oxidation is rarely experimentally reported for these materials. Furthermore, we found that atmosphere exposure enhanced SdH oscillation amplitudes and the carrier lift time, and the mobility increased by 30% after Sb_2_Se_2_Te surface oxidation.

## Conclusion

The non-saturating MR was studied in the Sb_2_Se_2_Te TI after different levels of N_2_ exposure. The MR slopes and the mobilities had the same dependency on temperature over a wide temperature range, that is consistent with the PL model of weak mobility fluctuations. All measured data conformed to a linear correlation between MR slope and mobility over a wide mobility range. The MR slope increased by approximately 100% after 2-days of N_2_ exposure at 10 K. On the other hand, the SdH oscillation amplitude increased by 30%, and the oscillation frequencies remained the same after N_2_ exposure. The carrier scattering time and mobility increased by 30%. These carrier transport characteristics enhancement might have been caused by a high-mobility 2DEG induced by the nitrogen adsorbed on the surface. XPS revealed an oxidation layer of thickness 2.8 nm on the crystal’s surface. In addition to exhibiting oxidation-resistant carrier transport, our results suggested that the adsorbed N_2_ may have optimized the carrier transport of the surface states in our Sb_2_Se_2_Te TI.
